# Jefferson Medical College

**Published:** 1843-01-21

**Authors:** H. T. Child


### PROFESSOR MÜTTER'S CLINIC.

*Dispensary of Jefferson Medical College, Jan.* 4, 1343.

(Reported by H. T. Child.)

The first patient introduced was a man æt. thirty, of sanguine temperament and feeble appearance, a shoemaker by trade, and a native of Philadelphia; and who several months before had suffered from a severe attack of acute conjunctivitis. This had resulted in corneal opacity—the whole cornea of the left eye, and nearly the whole of the right, being perfectly opaque. The upper third of the right cornea still retained its transparency. Professor M. deemed the case a very fair one for the operation of Cunier. This operation was designed for the purpose of restoring vision where the blindness depended on corneal opacity, and where the opaque spot covered the pupil so as to prevent the transmission of light, and where a portion of the cornea—to one side or the other, or above or below the opacity—retains its transparency.

It consists in displacing the ball from its natural position, by the section of one or more of its muscles, so as to produce a certain degree of squinting, by which change in the axis of the eye, the transparent portion of the cornea is brought opposite the pupil, and the patient at once regains a certain degree of vision, and, in some cases, the function is almost perfectly restored.

The position of the transparent spot indicates which muscle should be divided. In the patient, this being at the upper portion of the cornea, it became necessary to divide the *superior straight* muscle, which was accordingly done, and the inferior straight, its antagonist, drew the ball downwards, and thus brought the transparent spot opposite the pupil. When the outer portion of the cornea is sound, the *external* straight muscle must be divided, which will produce strabismus convergens; and when the cornea is transparent at its inner margin, we divide the internal straight, and produce strabismus divergens; the inferior straight should be divided when the lower portion of the cornea retains its transparency—the other portions being opaque.

The operation is the same as that practised for strabismus, *i. e.*, the conjunctiva is divided with the scissors, the muscles taken on a blunt hook, and then divided with the scissors. Prof. Mütter rarely uses the speculum in operating on the eye.

A man æt. twenty-eight, an Irishman by birth, and a common labourer, was next brought in. On examination it was found that he could scarcely articulate, in consequence of a congenital *tongue-tye*, which bound the organ down so closely that it could neither be protruded or placed against the roof of his mouth. Prof. M. remarked, that it was surprising, in these days of cutting and hacking, how this man had so long escaped the surgeon's knife. He also begged the class to recollect, that the operation about to be performed, was required by the malformation of the organ, and had been practised from time immemorial for the same defect. He deprecated most strongly any attempt being made to cure functional stammering by a similar operation; and declared that the experience of all surgeons went to prove the entire inutility of this measure. The frænum linguæ, and the attachment of the genio-hyo-glossus, were then divided with curved sharp-pointed scissors, and the patient at once protruded his tongue, and exhibited a manifest improvement in his speech. There still remained a stammer (the effect of long habit), which may continue, but generally it gradually disappears.

The next case was one of double hernia, the protrusions existing at the umbilicus and the right groin. The defect was congenital, and the patient a boy two months old. In cases of congenital umbilical hernia, Prof. M. has been in the habit, after reducing the hernia, of including the sac in a ligature, and thus, by causing adhesive inflammation in its walls, secures its obliteration. He cautioned the class against performing this operation in children who were much older than this, and in whom the external tunic of the hernia is composed of the common integument. In these the operation is exceedingly painful, and apt to bring on inflammation of the peritoneum; the same remark applies to adults. In these cases pressure, by a well-constructed truss, is our only remedy. As in the very young the external coat of the hernia is generally composed of the cellular tissue that holds the remains of the cord together, there is less pain and less risk of inflammation, in such cases, from the use of the ligature.

The operation was performed by placing the child on its back, returning the hernial contents by pressure, then passing a strong needle through the base of the sac, and tying a ligature beneath this firmly, so as to strangulate the parts completely. In a few minutes the pain passed off, and the child went to sleep. The scrotal hernia was not treated, the Dr. wishing to irritate the child as little as possible. A truss was ordered for it.

The fourth case was one of tertiary syphilis; the patient a female, æt. about 40, had suffered for a long time under this disease, and exhibited a most terrible example of its ravages; her skin was covered with the copper-coloured blotches; large ulcers covered her forehead, shoulders and ankles, and she suffered excruciating agony at night. Prof. M. dwelt upon the recent views entertained with respect to the nature of this complaint, and particularly those of Ricord, He ordered for the patient a mild and nutritious diet—the

*iodide of potassium*, in five-grain doses, to be taken three times a day—the syrup of sarsaparilla, in table spoonful doses, and aromatic wine as a dressing for the ulcers—the wine to be applied on lint, and the dressing to be covered with oiled silk to prevent evaporation—the pulv. ipecac. comp., in 5 gr. doses, was also ordered, in case the patient did not sleep well at night.

There were fourteen other cases present, the most interesting of which were: caries of lower jaw, tumour of the face, false anchylosis of elbow-joint, acute conjunctivitis, scrofula, and chronic disease of testis.

